# Supplementary material for: Physical function, daily activities, and spinal pain in the elderly: A cross-sectional study
Source: PLoS One. 2026 Mar 10;21(3):e0344292. doi: 10.1371/journal.pone.0344292 (PMC12974841; doi:10.1371/journal.pone.0344292)
Supplement: Attachment — Submitted filename: response_reviewers_editor__.docx [file pone.0344292.s001.docx]

**Response to the Academic Editor and Reviewers**

| Manuscript title: | Physical function, daily activities, and spinal pain in the elderly: A cross-sectional study |
| --- | --- |
| Journal: | PLOS ONE |
| Manuscript ID: | PONE-D-25-51057 |
| Corresponding author: | Paulina Kowalewska |

**Dear Academic Editor, Dear Reviewers,**

We thank you for your careful evaluation of our manuscript and for the constructive comments. We have revised the manuscript thoroughly to improve clarity, reporting quality, and the interpretation of our findings. A tracked-changes version and a clean version have been submitted, as requested.

# Summary of major revisions

- Expanded the Discussion with a dedicated mechanistic subsection linking cervical pain, postural control, and physical activity behavior.
- Updated the Introduction with more recent epidemiological and public health evidence; improved transitions and made the research gap and hypotheses explicit.
- Clarified study design (cross-sectional, STROBE-aligned), recruitment, eligibility criteria, and testing procedures.
- Moved and expanded Table 1 (participant characteristics + outcomes) to Results; removed interpretive wording from Results.
- Replaced multiple correlation figures with a single Spearman correlation matrix (Table 2) and reconciled all r and p values.
- Added a concise paragraph on psychometric properties and measurement considerations for all tools (including known limitations such as IPAQ overestimation).
- Strengthened limitations (cross-sectional design, sample size, lack of covariate adjustment, and the absence of dedicated gait-speed metrics).
- Checked formal requirements (ethics placement, ORCID, figure/table cross-references, data availability DOI).

# 1. Academic Editor

**AE-1**

Comment: *“mechanisms underlying the links between cervical spine pain and postural disorders, and therefore the impact on physical activity levels, are insufficiently explained.”*

**Response:**

We agree and have expanded the Discussion to provide a clearer, evidence-based mechanistic rationale linking cervical pain to postural control disturbances and, downstream, to potential reductions in physical activity.

- Added a distinct mechanistic subsection describing how cervical pain and sensorimotor deficits can impair cervical proprioception and multisensory integration (cervical-visual-vestibular), contributing to increased postural sway and fall risk.
- Explicitly connected this framework to our data, including the observed association between neck disability and COP sway area.
- Clarified the plausible behavioral pathway to physical activity (pain-related avoidance, reduced confidence, and fear of falling), while emphasizing that our cross-sectional findings support associations rather than causation.
- Strengthened the interpretation of the IPAQ results by acknowledging self-report limitations and explaining how these limitations may influence observed relationships.

Overall, these additions ensure that cited studies are directly linked to our specific outcomes and that the mechanistic argument is presented as an explanatory model, not a causal conclusion.

**Manuscript changes:**

Discussion: new/expanded mechanistic subsection (cervical pain - sensorimotor deficits - postural sway) and an explicit paragraph linking the framework to physical activity behavior and IPAQ interpretation; Limitations: strengthened language on cross-sectional design and self-report physical activity.

# 2. Reviewer 1

**R1-1**

Comment: *The reviewer considers the work interesting and valid but asks for clearer mechanisms linking neck pain to postural disorders and physical activity, with updated references.*

**Response:**

We thank the reviewer. Our revisions are aligned with the response to the Academic Editor and focus on strengthening the mechanistic explanation and ensuring that the literature is current and directly relevant to our findings.

- Expanded the Discussion with a structured description of the cervical sensorimotor deficit model in older adults.
- Updated and integrated key mechanistic references and linked them explicitly to our outcomes (notably the NDI - COP sway area association).
- Clarified the plausible connection to physical activity behavior and framed it cautiously (association, not causation).

**Manuscript changes:**

Discussion: mechanistic subsection expanded and updated; Introduction/Discussion: references refreshed where prior citations were outdated.

# 3. Reviewer 2

## 3.1 Introduction

**R2-1**

Comment: *Some cited literature (particularly public health) is older than 10 years and should be updated.*

**Response:**

We updated the Introduction to include more recent evidence (including population-based studies and meta-analytic work) on:

- population aging trends;
- the epidemiology of spinal pain (cervical and lumbar);
- the relationship between physical activity, functional fitness, and health in older adults.

Older references were retained only when they represent foundational work, and they are now complemented by current sources.

**Manuscript changes:**

Introduction: updated epidemiology/public-health background paragraphs and updated reference list.

**R2-2**

Comment: *Lack of transitions.*

**Response:**

We improved the flow of the Introduction by adding clear transitional sentences:

- between the physical activity background and the introduction of pain as a barrier to movement;
- between risk-factor discussion and aging/fitness context;
- between the background and the study aim;
- between the aim and hypotheses.

**Manuscript changes:**

Introduction: revised transitions and reorganized paragraph order for clearer logic.

**R2-3**

Comment: *Scientific justification / research gap not clearly articulated.*

**Response:**

We rewrote the final part of the Introduction to make the research gap explicit and to clarify why this study adds value. In particular, we now emphasize the limited number of studies that simultaneously evaluate spinal pain (cervical and lumbar) alongside balance, mobility, reaction time, step length, muscle strength, and physical activity in community-dwelling older adults.

**Manuscript changes:**

Introduction: revised final paragraph (research gap) and strengthened study rationale.

**R2-4**

Comment: *Hypotheses not explicitly stated in the article.*

**Response:**

We now state the hypotheses explicitly at the end of the Introduction. We also adjusted wording throughout the manuscript to refer to results as being 'consistent with' hypotheses rather than 'confirming' them.

**Manuscript changes:**

Introduction: hypotheses added explicitly; Discussion: wording adjusted to avoid confirmatory/causal language.

## 3.2 Methods

**R2-5**

Comment: *Study design is not reported.*

**Response:**

We now explicitly state at the beginning of the Methods that this is an observational, cross-sectional study, and we note that reporting follows STROBE guidance.

**Manuscript changes:**

Methods: opening paragraph updated to specify study design and reporting standard.

**R2-6**

Comment: *Participant recruitment procedure is not described.*

**Response:**

We expanded the Participants subsection to detail recruitment channels (e.g., senior clubs, local community announcements) and the screening approach used to verify eligibility.

**Manuscript changes:**

Methods - Participants: recruitment and screening details added.

**R2-7**

Comment: *Table with characteristics belongs to Results and should include all outcomes.*

**Response:**

We moved the participant characteristics table to the Results section and expanded it into a comprehensive Table 1 including demographics and all clinical/functional outcomes.

**Manuscript changes:**

Results: Table 1 placed in Results and expanded (demographics + all outcomes).

**R2-8**

Comment: *Exclusion criteria vs non-inclusion terminology.*

**Response:**

We standardized terminology throughout the manuscript by using only 'inclusion criteria' and 'exclusion criteria' and removed the ambiguous term 'non-inclusion'.

**Manuscript changes:**

Methods: eligibility terminology standardized and clarified.

**R2-9**

Comment: *Order of inclusion/exclusion criteria vs protocol description.*

**Response:**

We reorganized the Methods so that eligibility criteria are presented before the detailed testing protocol and procedures.

**Manuscript changes:**

Methods: reordered subsections for clearer readability.

**R2-10**

Comment: *Psychometric properties of tests and questionnaires are missing.*

**Response:**

We added a dedicated paragraph in the Methods summarizing validity/reliability and key measurement considerations for each tool. In addition, we acknowledged known limitations where relevant (e.g., self-report bias and overestimation in IPAQ).

**Manuscript changes:**

Methods: new paragraph summarizing psychometric properties/measurement considerations for NDI/ODI, IPAQ, TUG, posturography outcomes, dynamometry, and reaction time testing.

**R2-11**

Comment: *Postural stability outcome: mean sway velocity vs sway area.*

**Response:**

We agree that mean sway velocity is an informative parameter. In this study we used sway area (ellipse area) as the primary stability outcome based on its interpretability and the available reliability evidence in older adults. We now justify this choice explicitly and list the absence of velocity measures as a limitation and recommendation for future work.

**Manuscript changes:**

Methods: rationale for sway area added; Discussion/Limitations: explicit note on omission of sway velocity and recommendation for future studies.

**R2-12**

Comment: *TUG does not measure gait speed; 10 m walk test is essential.*

**Response:**

We agree that TUG reflects functional mobility and dynamic balance rather than isolated gait speed. We clarified this in the Methods and removed statements implying that TUG measures gait speed. We also acknowledge the absence of a dedicated gait-speed test (e.g., 10 m walk test) as a limitation.

**Manuscript changes:**

Methods: clarified construct measured by TUG; Limitations: absence of 10 m walk test/gait-speed metric noted.

**R2-13**

Comment: *Visuomotor reaction test: provide more information on validity in older adults.*

**Response:**

We added a clearer description of the reaction-time assessment (task, outcome definition, and device/system used). Where validation evidence in older adults is available, we cite it; where evidence is limited, we acknowledge this explicitly as a measurement limitation.

**Manuscript changes:**

Methods: expanded reaction-time testing description; Limitations: clarified evidence base and potential measurement constraints.

**R2-14**

Comment: *No sample size calculation / power analysis.*

**Response:**

We acknowledge that no a priori sample-size calculation was conducted. To address the concern without overstating the role of post-hoc calculations, we added a brief sensitivity statement indicating the approximate correlation magnitude that can be detected with N = 28 at alpha = 0.05 (two-tailed). We also strengthened the limitations regarding generalizability.

**Manuscript changes:**

Statistical analysis: added sensitivity/power statement; Limitations: expanded sample-size/generalizability limitation.

**R2-15**

Comment: *No multivariate analysis / no adjustment for covariates.*

**Response:**

We agree that covariate-adjusted models would be valuable. However, with N = 28, multivariable regression with several predictors/covariates would be statistically unstable and at high risk of overfitting. We therefore limited analyses to bivariate correlations and clearly stated this rationale, while acknowledging it as a limitation.

**Manuscript changes:**

Statistical analysis: clarified rationale for bivariate approach; Limitations: lack of covariate adjustment explicitly noted.

## 3.3 Results

**R2-16**

Comment: *A full descriptive section of the study population is required.*

**Response:**

We added a descriptive paragraph summarizing participant characteristics and direct references to the comprehensive Table 1.

**Manuscript changes:**

Results: descriptive paragraph added; Table 1 referenced appropriately.

**R2-17**

Comment: *Results contain interpretations; move to Discussion.*

**Response:**

We removed interpretive language (e.g., 'this suggests', 'indicating that') from the Results. The Results now report data only; interpretations were moved to the Discussion.

**Manuscript changes:**

Results: interpretive phrasing removed; Discussion: interpretation consolidated.

**R2-18**

Comment: *Relevance of correlations (age-step length, etc.) to the main research question.*

**Response:**

We clarified that correlations involving age (e.g., age vs step length/reaction time) are secondary analyses intended to characterize the functional profile of the sample rather than the primary focus of the pain analysis.

**Manuscript changes:**

Introduction/Discussion: clarified primary vs secondary aims and the role of age-related correlations.

**R2-19**

Comment: *Inconsistencies between r and p values in text vs figures.*

**Response:**

We verified all calculations and ensured that all r and p values are consistent across the text and the revised correlation table. Figures that previously contained inconsistencies were removed.

**Manuscript changes:**

Results: values harmonized with Table 2; figures removed and cross-references cleaned.

**R2-20**

Comment: *No summary table of correlations; seven figures instead.*

**Response:**

We removed the multiple correlation figures and replaced them with a single Spearman correlation matrix (Table 2), which presents the results more transparently.

**Manuscript changes:**

Results: Figures removed; Table 2 added and cited in the text.

## 3.4 Discussion

**R2-21**

Comment: *Correlations described as causal.*

**Response:**

We agree this is important. We carefully revised the Discussion to remove causal wording (e.g., 'influences', 'affects') and replaced it with associative language (e.g., 'associated with', 'co-occurs with', 'consistent with'). We also added an explicit statement that the findings represent associations, not causal effects.

**Manuscript changes:**

Discussion: causal language removed; explicit association-only statement added.

**R2-22**

Comment: *Long paragraphs with references not clearly linked to the findings.*

**Response:**

We streamlined the Discussion by splitting long paragraphs and ensuring that each cited study is directly tied to our specific results or to the mechanistic explanation of those results.

**Manuscript changes:**

Discussion: paragraph structure improved; references better aligned with study findings.

## 3.5 Editorial and formal requirements

We addressed all remaining editorial and formal requirements as follows:

- Adjusted manuscript structure and file naming to match journal templates.
- Ensured the ethics statement is placed exclusively in the Methods section.
- Updated the corresponding author's ORCID in the submission system.
- Removed references to deleted figures and ensured new tables are correctly cited.
- Provided a direct DOI to the public repository for data availability.
- Verified the reference list (including adding suggested literature and checking for retractions, where applicable).
- Revised the manuscript for clarity and standard English usage (grammar and typography).

We again thank the Academic Editor and Reviewers for their time and guidance. We hope the revised manuscript adequately addresses all concerns and is now suitable for publication.

Sincerely,

Paulina Kowalewska, on behalf of all authors
