# Supplementary material for: Physical function, daily activities, and spinal pain in the elderly: A cross-sectional study
Source: PLoS One. 2026 Mar 10;21(3):e0344292. doi: 10.1371/journal.pone.0344292 (PMC12974841; doi:10.1371/journal.pone.0344292)
Supplement: Attachment — Submitted filename: Response to Reviewers.docx [file pone.0344292.s002.docx]

We would like to thank the Academic Editor and the Reviewers for their careful evaluation of our manuscript and for the constructive comments provided.

We have addressed all remaining minor comments as follows:

1. **Abstract**
   A brief introductory sentence has been added at the beginning of the abstract, prior to the statement of the study objective, as requested.
2. **Figures and tables in the Results section**
   In accordance with the previous recommendations and to improve clarity and readability, the figures in the Results section have been replaced with tables. The relevant results are now presented in tabular form and are explicitly referenced in the main text.

We believe that these revisions ensure full compliance with the journal’s presentation guidelines.
We appreciate the positive assessment of our work and hope that the revised manuscript is now suitable for publication in PLOS ONE.

**Kind regards,
Paulina Kowalewska**
